# Online measurement method for dimensions of disk parts based on machine vision

**DOI:** 10.1371/journal.pone.0307525

**Published:** 2024-07-25

**Authors:** Jianwei Miao, Qingchang Tan, Baorui Sun, Jinghe Zhao, Siyuan Liu, Yongqi Zhang

**Affiliations:** 1 School of Mechanical Engineering, Changchun Guanghua University, Changchun, China; 2 School of Mechanical and Aerospace Engineering, Jilin University, Changchun, China; 3 Sinotest Equipment Co., Ltd., Changchun, China; 4 Army Academy of Armored Forces, Changchun, China; National Institute of Laser Enhanced Sciences (NILES), Cairo University, EGYPT

## Abstract

Online measurement of disk part dimensions by the standard industrial camera features low cost, high efficiency and good universality, but the impact of projection distortion and end face chamfer on measurement is needed to overcome. Present work presents a measurement method to resolve above issues based on machine vision. To improve the measurement accuracy, lower end face of a disk part is determined as calibration plane and the upper end face is measurement plane. To reduce the impact of projection distortion and chamfer on measurement, the measurement points are reconstructed on the measurement plane by re-projection. Then, the inner and outer diameters of disk parts are measured by circle geometric fitting, and the thickness is calculated by the measurement plane position. The experimental results show that the method can online measure disk part dimensions just by a single image, and accuracy meets the requirements of universal grade disk parts.

## 1 Introduction

Disk parts are important mechanical components that are widely used in industry [1]. Online measurement technology based on machine vision is of great significance for development of intelligent and digital manufacturing technology [2,3], and has been widely studied in recent years [4,5].

Wen et al. [6] provided a method for detecting bearing roller defects based on machine vision. Shen et al. [7] developed an automatic detection system for flange surface defects based on machine vision, which can achieve surface defect test with a minimum width of 0.1 mm by parallel light projection. A telecentric lens was used to detect industrial gear parameters, and the Vision2D measurement system was developed to improve detection accuracy, reduce downtime, and optimize the detection process in [8]. A surface defects inspection system based on machine vision for clutch friction disks was developed to measure its shape and surface size in [9]. Wang et al. [10] proposed a visual measurement method for gear parameters, which can measure the root and top circle diameters of gears.

The above papers have conducted in-depth research on visual measurement of disk parts, but most of them focus on surface inspection. At present, no research has been found on simultaneous online visual measurement of inner diameter, outer diameter, and thickness of disk parts. Furthermore, some studies [11–13] use telecentric lenses to improve measurement accuracy, but telecentric lenses with high cost, small field of view, low measurement efficiency, and low universality, which make it difficult to satisfy the requirements of in-situ applications.

This work uses a standard industrial camera to realize high-precision online measurement of inner, outer diameters and thicknesses of disk parts just by a single image. In order to facilitate online measurement, take upper end face of the disk part (abbreviate as upper end face) as the measurement plane, and take points on the edge of the disk part as the measurement points for inner, outer diameters and thickness (abbreviate as measurement points). To eliminate the influence of the projection distortion on the measurement, reconstruct three-dimensional coordinates of the measurement points so that inner, outer diameters and thickness of the parts can be calculated on the measurement plane. Then, correct three-dimensional coordinates of the measurement points at chamfer. Finally, compare the measurement results obtained from the experiment with those obtained by a micrometer to verify accuracy of the measurement method. This work has five sections. Section 2 is camera calibration and coordinates transformation. Section 3 measures dimensions of disk parts. Section 4 experimentally tests the measurement method and section 5 provides conclusions of the study.

## 2 Camera calibration and coordinates transformation

### 2.1 Camera calibration and image processing

**Fig 1:**
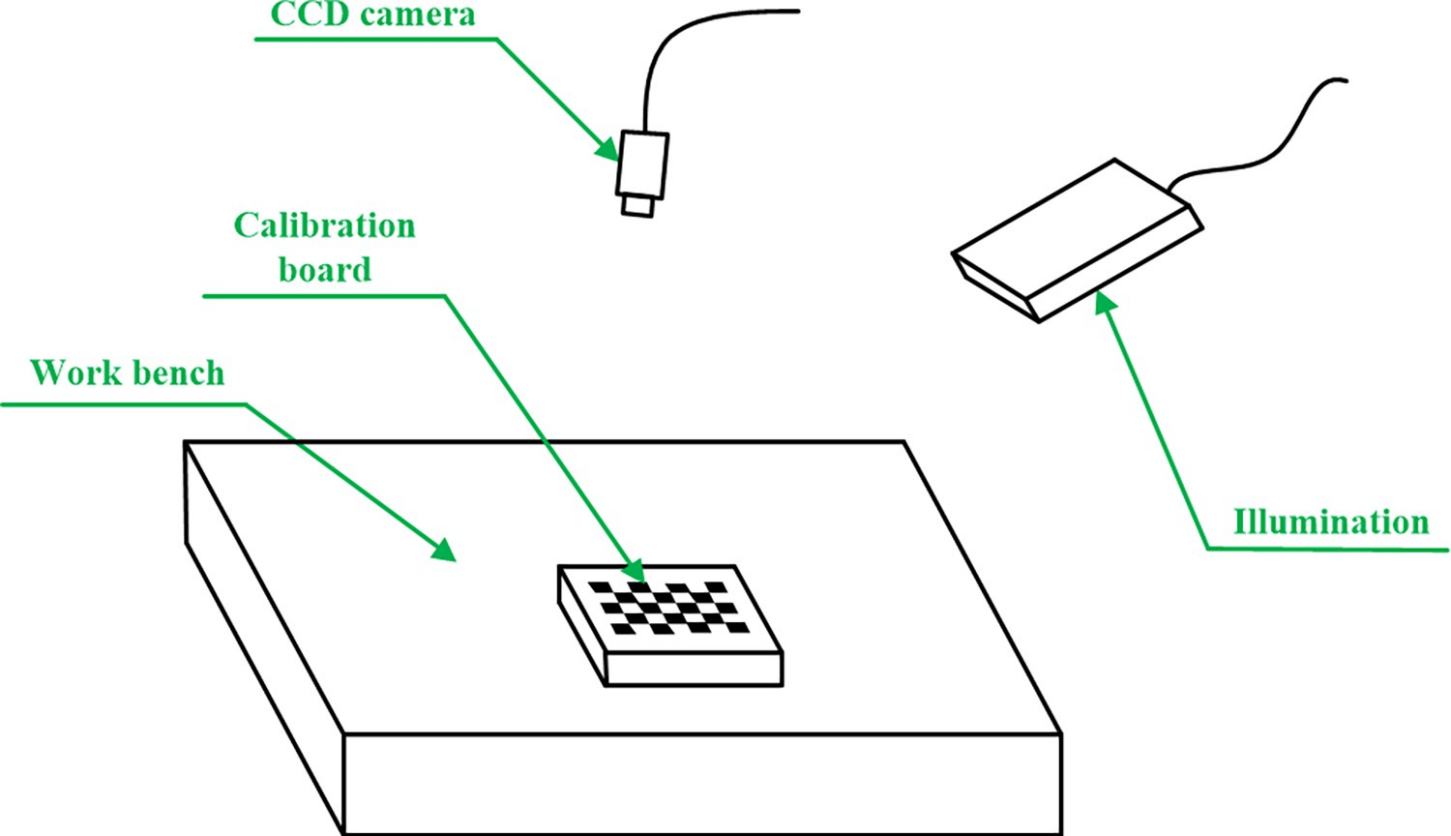
Calibration of camera extrinsic parameters.

Camera intrinsic parameters are calibrated by checkerboard calibration board [14]. The part is placed on a workbench for visual measurement, so the workbench plane (that is lower end face of the part) is determined as calibration plane of camera extrinsic parameters, as shown in Fig 1. Equation of the part lower end face is calculated by calibrated equation of the checkerboard plane and known thickness of the calibration board. To see Appendix A in S1 Appendix for details of calibration results of camera intrinsic and extrinsic parameters.

The noise on machine vision images is usually Gaussian noise caused by accidental factors [15], so Gaussian filtering algorithm is used to filter and denoise the image. Balancing the speed of image processing and the enhancement effect of image, the Retinex algorithm based on center wrapping is adopted for image enhancement processing [16]. To improve the efficiency of feature points extraction, a grayscale template matching algorithm is used to determine the ROI (region of interest) on the image [17]. Sub pixel positions of the edge points on image are extracted by the method based on Zernike moment [18], and the singularities from feature points are removed by the Laida Criterion.

When disk parts are placed on the workbench for visual measurement, edges of the part upper end face on the image are more apt to detect, so the part upper end face is set as measurement plane.The difference in thickness of disk parts makes the difference in position of upper end face, so after calibrating the part lower end face (which coincides with the workbench plane) as the camera extrinsic parameters, the position of upper end face (that is measurement plane) is determined by perspective projection transformation and the known part chamfer size.

### 2.2 Coordinate transformation

**Fig 2:**
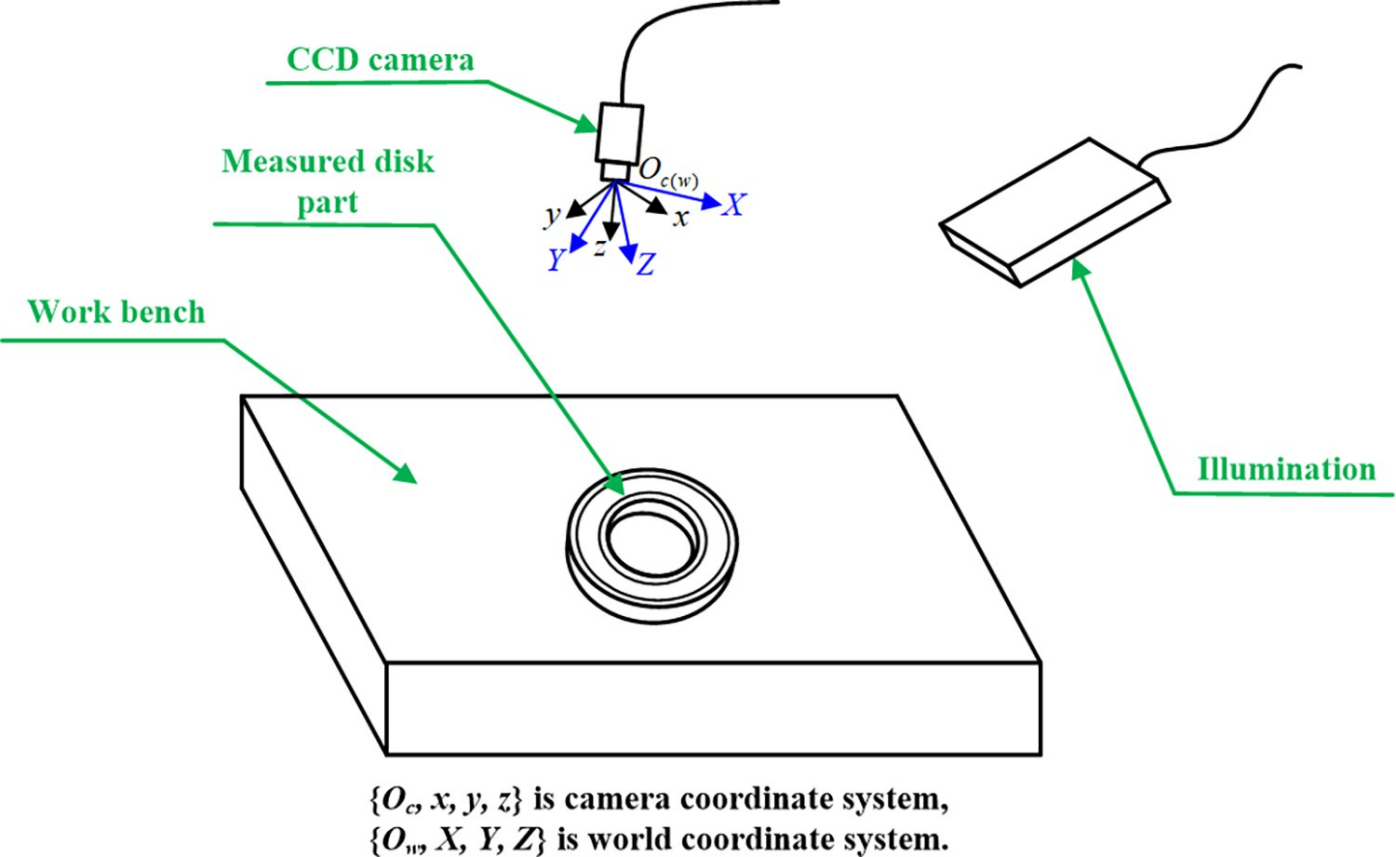
Measurement for dimensions of disk parts based on machine vision.

As shown in Fig 2, origin of the world coordinate system is set at that of the camera coordinate system, that is, it coincides with the optical center of camera. The *Z*-axis of world coordinate system points vertically towards the workbench plane. Set a axis ***V***, which passes through the origin and follows the cross product direction of *z*-axis of the camera coordinate system and *Z*-axis of the world coordinate system. Rotate the *z*-axis around ***V*** to the position coincided with the *Z*-axis, and then directions of the *x*-axis and *y*-axis of camera coordinate system after rotation are defined as directions of the *X*-axis and *Y*-axis of world coordinate system.

In camera coordinate system, equation of the part lower end face obtained by calibration is

$$A_{0}x+B_{0}y+C_{0}z+1=0$$ (1)

The normal vector of lower end face is (*A*_0_,*B*_0_,*C*_0_), then directional cosine of the *Z*-axis of world coordinate system ***Z***_***k***_ is

$$Z_{k}={\left(i_{13},i_{23},i_{33}\right)}^{T}={\left(-\frac{A_{0}}{\sqrt{A_{0}^{2}+B_{0}^{2}+C_{0}^{2}}},-\frac{B_{0}}{\sqrt{A_{0}^{2}+B_{0}^{2}+C_{0}^{2}}},-\frac{C_{0}}{\sqrt{A_{0}^{2}+B_{0}^{2}+C_{0}^{2}}}\right)}^{T}$$ (2)

Directional cosine of the *z*-axis of camera coordinate system is ***z***_***k***_ = (0,0,1)^*T*^, and the directional vector ***v***_***d***_ of axis ***V*** is the cross product of ***Z***_***k***_ and ***z***_***k***_.


$$v_{d}=z_{k}\times Z_{k}={\left(-i_{23},i_{13},0\right)}^{T}$$ (3)

Unitizing ***v***_***d***_ to obtain the directional cosine ***v***_***k***_ of axis ***V***.


$$v_{k}={\left(\frac{-i_{23}}{\sqrt{i_{13}^{2}+i_{23}^{2}}},\frac{i_{13}}{\sqrt{i_{13}^{2}+i_{23}^{2}}},\;0\right)}^{T}$$ (4)

The cross product matrix ***K*** of ***v***_***k***_ is

$$K=[\begin{array}{ccc} 0 & 0 & \frac{i_{13}}{\sqrt{i_{13}^{2}+i_{23}^{2}}}\\ 0 & 0 & \frac{i_{23}}{\sqrt{i_{13}^{2}+i_{23}^{2}}}\\ -\frac{i_{13}}{\sqrt{i_{13}^{2}+i_{23}^{2}}} & -\frac{i_{23}}{\sqrt{i_{13}^{2}+i_{23}^{2}}} & 0 \end{array}]$$ (5)

Directional cosine of the *z*-axis of camera coordinate system is ***z***_***k***_ =(0, 0, 1)^*T*^, and that of the *Z*-axis of world coordinate system is ***Z***_***k***_ = (*i*_13_, *i*_23_, *i*_33_)^*T*^. Their included angle *β*_*R*_ is

$$\beta_{R}=\arccos(i_{33})$$ (6)

According to the Rodrigues rotation transformation [19], combined with Eqs (5) and (6), the rotation matrix ***R*** of *z*-axis and *Z*-axis can be obtained.

$$R=[I_{one}+(1-\cos\beta_{R})K^{2}+\sin\beta_{R}K]$$ (7)

Where ***I***_***one***_ is identity matrix.

When the *z*-axis of camera coordinate system is rotated to the position coincided with the *Z*-axis of world coordinate system, positions of the *x*-axis and *y*-axis of camera coordinate system after rotation are those of the *X*-axis and *Y*-axis of world coordinate system. According to directional cosines ***x***_***k***_ = (1, 0, 0)^*T*^ and ***y***_***k***_ = (0, 1, 0)^*T*^ of the x-axis and y-axis of camera coordinate system, those of the *X*-axis and *Y*-axis of world coordinate system in camera coordinate system can be obtained by Eq (7).


$$X_{k}={\left(i_{11},i_{21},i_{31}\right)}^{T}=Rx_{k}={\left(1-\frac{i_{13}^{2}}{1+i_{33}},-\frac{i_{13}i_{23}}{1+i_{33}},-i_{13}\right)}^{T}$$ (8)

$$Y_{k}={\left(i_{12},i_{22},i_{32}\right)}^{T}=Ry_{k}={\left(-\frac{i_{13}i_{23}}{1+i_{33}},1-\frac{i_{23}^{2}}{1+i_{33}},-i_{23}\right)}^{T}$$ (9)

Set camera coordinates of the point to (*x*,*y*,*z*), and its world coordinates to (*X*,*Y*,*Z*). Then the transformation relationship between two coordinate systems can be expressed as

$$\left[\begin{array}{l} x\\ y\\ z \end{array}]=I[\begin{array}{c} X\\ Y\\ Z \end{array}]=[\begin{array}{ccc} i_{11} & i_{12} & i_{13}\\ i_{21} & i_{22} & i_{23}\\ i_{31} & i_{32} & i_{33} \end{array}][\begin{array}{c} X\\ Y\\ Z \end{array}]=[\begin{array}{ccc} 1-\frac{i_{13}^{2}}{1+i_{33}} & -\frac{i_{13}i_{23}}{1+i_{33}} & i_{13}\\ -\frac{i_{13}i_{23}}{1+i_{33}} & 1-\frac{i_{23}^{2}}{1+i_{33}} & i_{23}\\ -i_{13} & -i_{23} & i_{33} \end{array}][\begin{array}{c} X\\ Y\\ Z \end{array}\right]$$ (10)

The transformation matrix ***I*** can be determined based on the lower end face equation, as shown in Eq (1), obtained by extrinsic parameter calibration. Calculate camera coordinates of the intersection point *P*_0_ between the *z*-axis of camera coordinate system and the lower end face equation, and then convert them into world coordinates by Eq (10), the *Z* world coordinate *Z*_0_ of the point on lower end face can be obtained. To see Appendix B in S1 Appendix for details.

## 3 Visual measurement of disk parts dimensions

### 3.1 Reduce the impact of projection distortion on measurement accuracy

Projection distortion is mainly caused by the measured part surface not being parallel to the image plane. Therefore, the coordinates of detected points on the image are converted to the measurement plane (parallel to the measured part surface) for three-dimensional reconstruction, and then the part dimensions is calculated, which can effectively reduce the influence of projection distortion on measurement accuracy. To determine the position of measurement plane (upper end face), a projection plane is established, which coincides with the *Z* = 1 plane in world coordinate system and is parallel to measurement plane.

#### 3.1.1 Calculate diameters of circles on both sides of the part chamfer by geometrically fitting circles on the projection plane

In camera coordinate system, let the detection coordinates of measurement points on theoretical image plane be (*x*_*Li*_,*y*_*Li*_,1). According to Eq (10), they can be transformed into world coordinates (*X*_*Li*_,*Y*_*Li*_,*Z*_*Li*_). Then the world coordinates (*X*_*Ti*_,*Y*_*Ti*_,*Z*_*Ti*_) of measurement points on projection plane can be obtained by perspective projection transformation, as shown in Eq (11).


$$X_{Ti}=\frac{X_{Li}}{Z_{Li}},\;Y_{Ti}=\frac{Y_{Li}}{Z_{Li}},\;Z_{Ti}=1$$ (11)

In world coordinate system, edges on both sides of the part chamfer on projection plane are circles. According to Eq (11) and the least squares method [20,21], the geometric fitting function of the circle is established.

$$\mathrm{Min}F=\sum_{i=1}^{n}\left[{\left(X-X_{Ti}\right)}^{2}+{\left(Y-Y_{Ti}\right)}^{2}\right]$$ (12)

Where (*X*,*Y*,1) are world coordinates of the point on the circle to be fitted that corresponds perpendicular to the measurement point (*X*_*Ti*_,*Y*_*Ti*_,1).

In world coordinate system, the circle equation to be fitted can be expressed as

$$\{\begin{array}{c} {\left(X-X_{c}\right)}^{2}+{\left(Y-Y_{c}\right)}^{2}-r_{t}^{2}=0\\ Z=1 \end{array}$$ (13)

Where (*X*_*c*_,*Y*_*c*_,1) are world coordinates of the circle center, and *r*_*t*_ is the circle radius

Expand the circle equation in Eq (13) into a general form, we can get

$$X^{2}+Y^{2}-2X_{c}X-2Y_{c}Y+X_{c}^{2}+Y_{c}^{2}-r_{t}^{2}=0$$ (14)

According to the geometric relationship [22,23] between (*X*_*Ti*_,*Y*_*Ti*_,1) and (*X*,*Y*,1), Eq (15) are established.


$$\begin{array}{l} f_{1}(X,Y)=X^{2}+Y^{2}-2X_{c}X-2Y_{c}Y+X_{c}^{2}+Y_{c}^{2}-r_{t}^{2}=0\\ f_{2}(X,Y)=(X-X_{Ti})(Y_{Ti}-Y_{c})-(X_{Ti}-X_{c})(Y-Y_{Ti})=0 \end{array}$$ (15)

The Jacobian matrix ***J***_*c*_ of points coordinates (*X*,*Y*) on the circle over coefficients (*X*_*c*_,*Y*_*c*_,*r*_*t*_) of the circle equation general form can be obtained by Eq (15).

$$C=(\begin{array}{cc} X-X_{c} & Y-Y_{c}\\ Y_{Ti}-Y_{c} & X_{c}-X_{Ti} \end{array})$$ (16)

$$B=(\begin{array}{ccc} X-X_{c} & Y-Y_{c} & r_{t}\\ Y_{Ti}-Y & X-X_{Ti} & 0 \end{array})$$ (17)

$$J_{c}=C^{-1}B$$ (18)

When fitting, the center coordinates and radius obtained from the circle algebraic fitting are used as the initial values for geometric fitting. Then, according to Eq (18), the Gauss Newton iteration method [24] is used to geometrically fit Eq (12), and the radii *r*_1_ and *r*_2_ of circles on both sides of the part chamfer on projection plane are obtained.

#### 3.1.2 Determine the position of measurement plane based on projection plane

**Fig 3:**
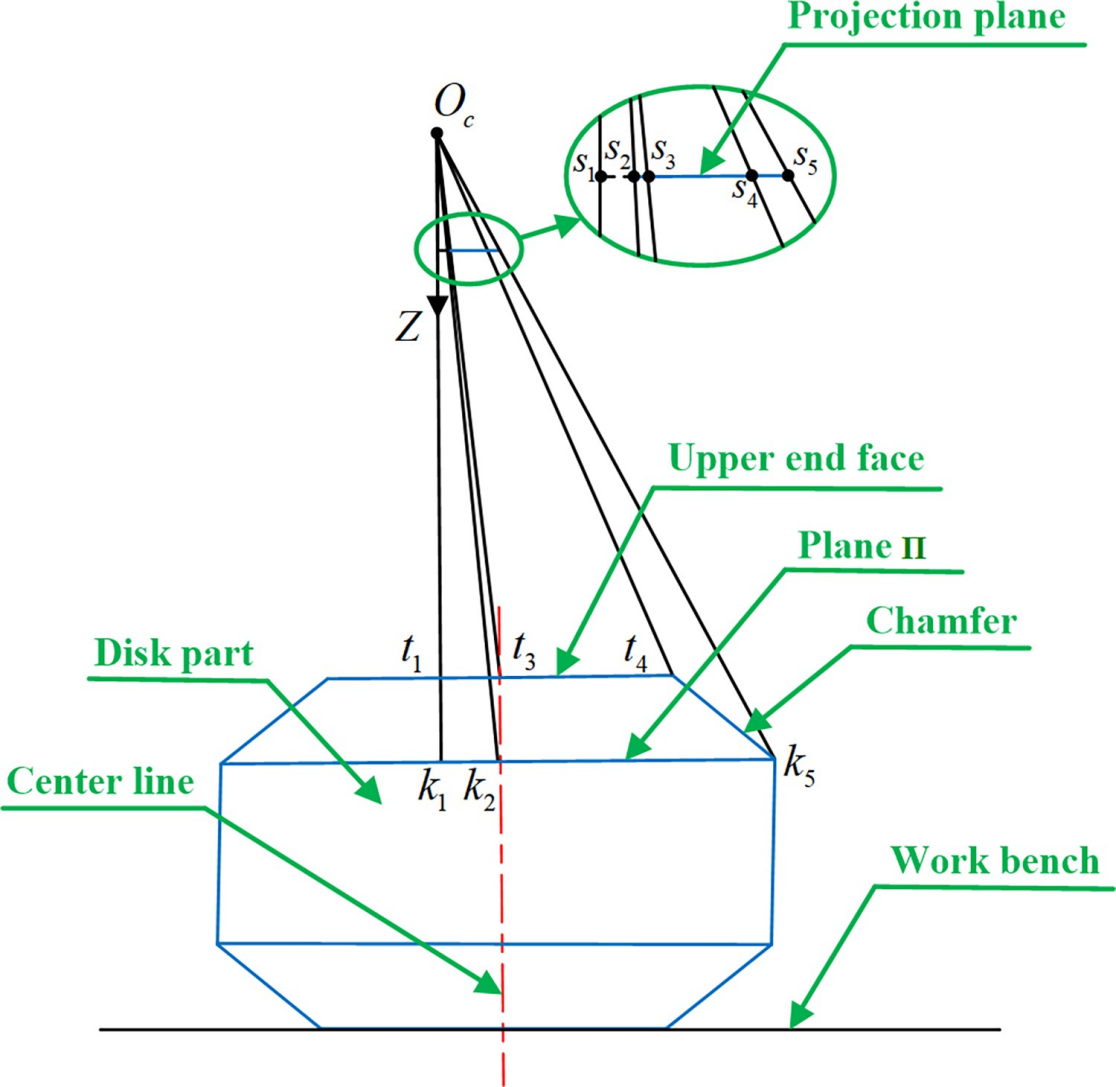
Determination of the upper end face position.

As shown in Fig 3, *O*_*c*_ is the camera optical center, and line segment *O*_*c*_*k*_1_ is on the *Z*-axis of world coordinate system. Points *s*_2_ and *s*_3_ are centers of the circles on both sides of the part chamfer fitted on projection plane, and the real centers corresponding to them are *k*_2_ and *t*_3_. Line segments *s*_3_*s*_4_ and *s*_2_*s*_5_ are the radii *r*_1_ and *r*_2_ of the circles fitted on projection plane, which are the perspective projections of line segments *t*_3_*t*_4_ on upper end face and *k*_2_*k*_5_ on plane Π respectively. Line segment *t*_3_*t*_4_ is radius *R*_1_ of the edge circle on upper end face, while line segment *k*_2_*k*_5_ is the true radius *R*_2_, which on plane II paralleled to upper end face. Since the projection plane is parallel to the upper end face, then $\Delta O_{c}s_{1}s_{3}∼\Delta O_{c}t_{1}t_{3}$, $\Delta O_{c}s_{3}s_{4}∼\Delta O_{c}t_{3}t_{4}$, we can get

$$\frac{O_{c}s_{1}}{O_{c}t_{1}}=\frac{O_{c}s_{3}}{O_{c}t_{3}}=\frac{s_{3}s_{4}}{t_{3}t_{4}}=\frac{1}{Z_{u}}=\frac{r_{1}}{R_{1}}$$ (19)

Where *Z*_*u*_ is the *Z* world coordinate of the point on measurement plane.

Similarly, $\Delta O_{c}s_{1}s_{2}∼\Delta O_{c}k_{1}k_{2}$, $\Delta O_{c}s_{2}s_{5}∼\Delta O_{c}k_{2}k_{5}$, and the length of line segment *t*_1_*k*_1_ is the known axial chamfer size *q*_1_ of the part, so

$$\frac{O_{c}s_{1}}{O_{c}k_{1}}=\frac{O_{c}s_{2}}{O_{c}k_{2}}=\frac{s_{2}s_{5}}{k_{2}k_{5}}=\frac{1}{Z_{u}+q_{1}}=\frac{r_{2}}{R_{2}}$$ (20)

According to the lengths of line segment *t*_3_*t*_4_ and *k*_2_*k*_5_, it can be obtained

$$R_{2}-R_{1}=q_{2}$$ (21)

Where *q*_2_ is the known radial chamfer size of the part, usually *q*_2_ = *q*_1_.

The *Z* world coordinate *Z*_*u*_ of the point on measurement plane can be determined by substituting Eqs (19) and (20) into Eq (21).

$$Z_{u}=\frac{q_{2}-r_{2}q_{1}}{r_{2}-r_{1}}$$ (22)

Where *Z*_*u*_ is a scalar, and its symbol is determined by the actual position of measurement plane relative to the *Z*-axis of world coordinate system.

#### 3.1.3 Reconstruct the world coordinates of measurement points on measurement plane

According to Eqs (11) and (22), the world coordinates (*X*_*ui*_,*Y*_*ui*_,*Z*_*ui*_) of measurement points on measurement plane are reconstructed by the ray tracing method, as shown in Eq (23).


$$X_{ui}=\frac{X_{Li}}{Z_{Li}}Z_{u},\;Y_{ui}=\frac{Y_{Li}}{Z_{Li}}Z_{u},\;Z_{ui}=Z_{u}$$ (23)

### 3.2 Reduce the impact of chamfer on measurement accuracy

Due to the part chamfer, the measurement points on measurement plane (upper end face of the part), as shown in Eq (23), are not the accurate measurement points for minimum inner diameter or maximum outer diameter of the part. The accurate measurement points should be on the edge of plane Π in Fig 3. Plane Π is parallel to the measurement plane, and the distance between two planes is known axial chamfer size *q*_1_ of the part, so the *Z* world coordinate of the point on plane Π is *Z*_Π_ = *Z*_*u*_+*q*_1_.

According to Eq (11), the world coordinates of measurement points on plane Π are reconstructed by the ray tracing method, as shown in Eq (24).


$$X_{\Pi i}=\frac{X_{Li}}{Z_{Li}}Z_{\Pi },\;Y_{\Pi i}=\frac{Y_{Li}}{Z_{Li}}Z_{\Pi },\;Z_{\Pi i}=Z_{\Pi }$$ (24)

### 3.3 Visual measurement of inner, outer diameters and thickness of disk parts

The circle geometric fitting model on plane Π can be established according to Eqs (12)–(18) and (18), and then the inner and outer diameters *d*_*n*_ and *d*_*w*_ of disk parts are obtained by geometrically fitting the circle on plane II.

In section 2.2, The *Z* world coordinate *Z*_0_ of the point on lower end face can be obtained by camera extrinsic parameters. So thickness *L*_*H*_ of the disk part is calculated by *Z*_0_ and Eq (22).


$$L_{H}=Z_{0}-Z_{u}$$ (25)

## 4 Experiment and analysis

**Fig 4:**
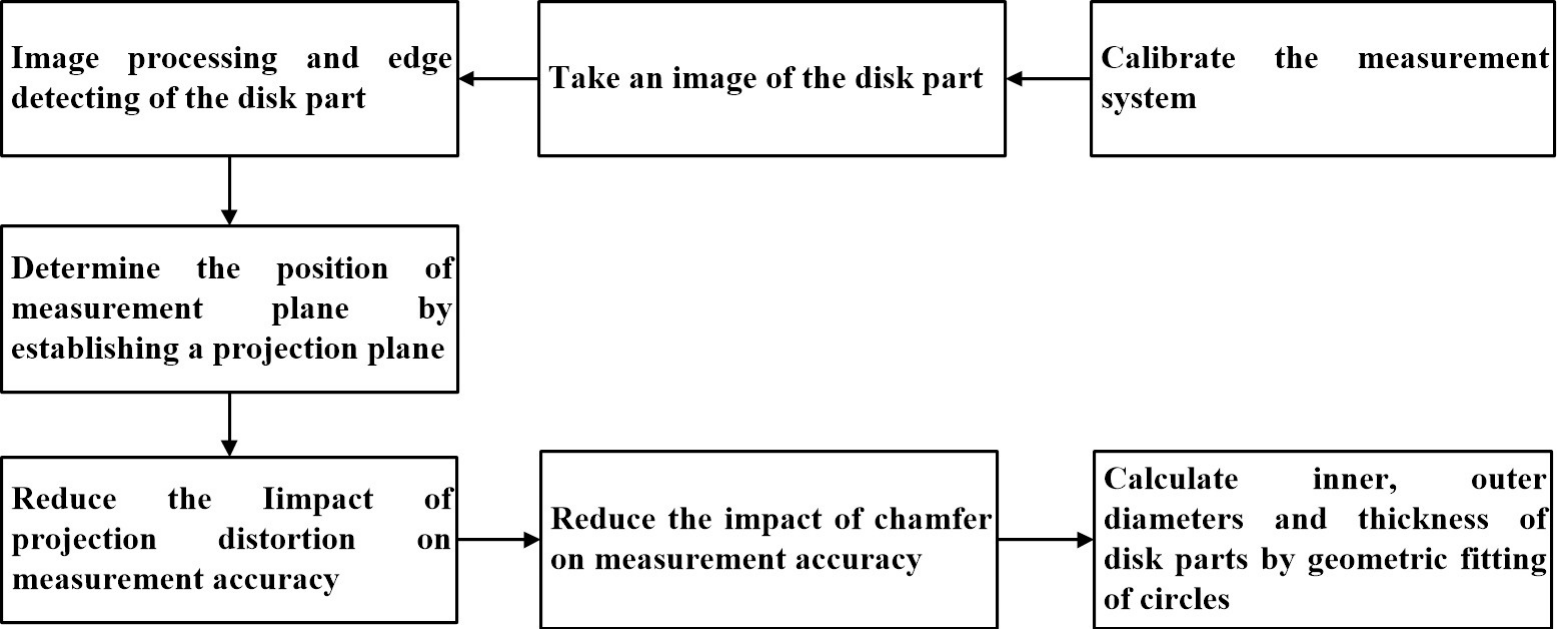
The flowchart for executing the experiment steps.

To verify accuracy of the online measurement method for the sizes of disk parts, a pulley, a flange, and a gear are selected for testing. The flowchart for executing the experiment steps is shown in Fig 4. The reference dimensions of three disk parts are obtained by a micrometer with an accuracy of ±0.001mm, the values are average of measuring 3 times, as shown in Table 1.

**Table 1:** Reference dimensions of disk parts for experiment (mm).

Disk Parts	Inner Diameters	Outer Diameters	Thicknesses	Chamfer Sizes
**Pulley**	13.016	63.126	19.961	C1
**Flange**	32.518	99.294	13.525	C1
**Gear**	31.620	113.360	27.463	C2

Table 2 shows main equipments and their parameters of the measurement system.

**Table 2:** Main parameters of the experimental equipments.

Equipments	Model No.	Main Parameters
**Computer**	Lenovo-M425-N007	Intel(R) Core(TM)-i5-8400
**CCD camera**	MV-CU120-10GM	Resolution: 4024×3036 pixel
**Lens**	MVL-KF2528M-2MPE	Focal length: 25 mm
**Calibration board**	ZNM01-63-2F	Precision of the grid: ±1.0–2.0μm
**Illumination**	LTS-LIP385-W	Working distance: 2000mm

**Fig 5:**
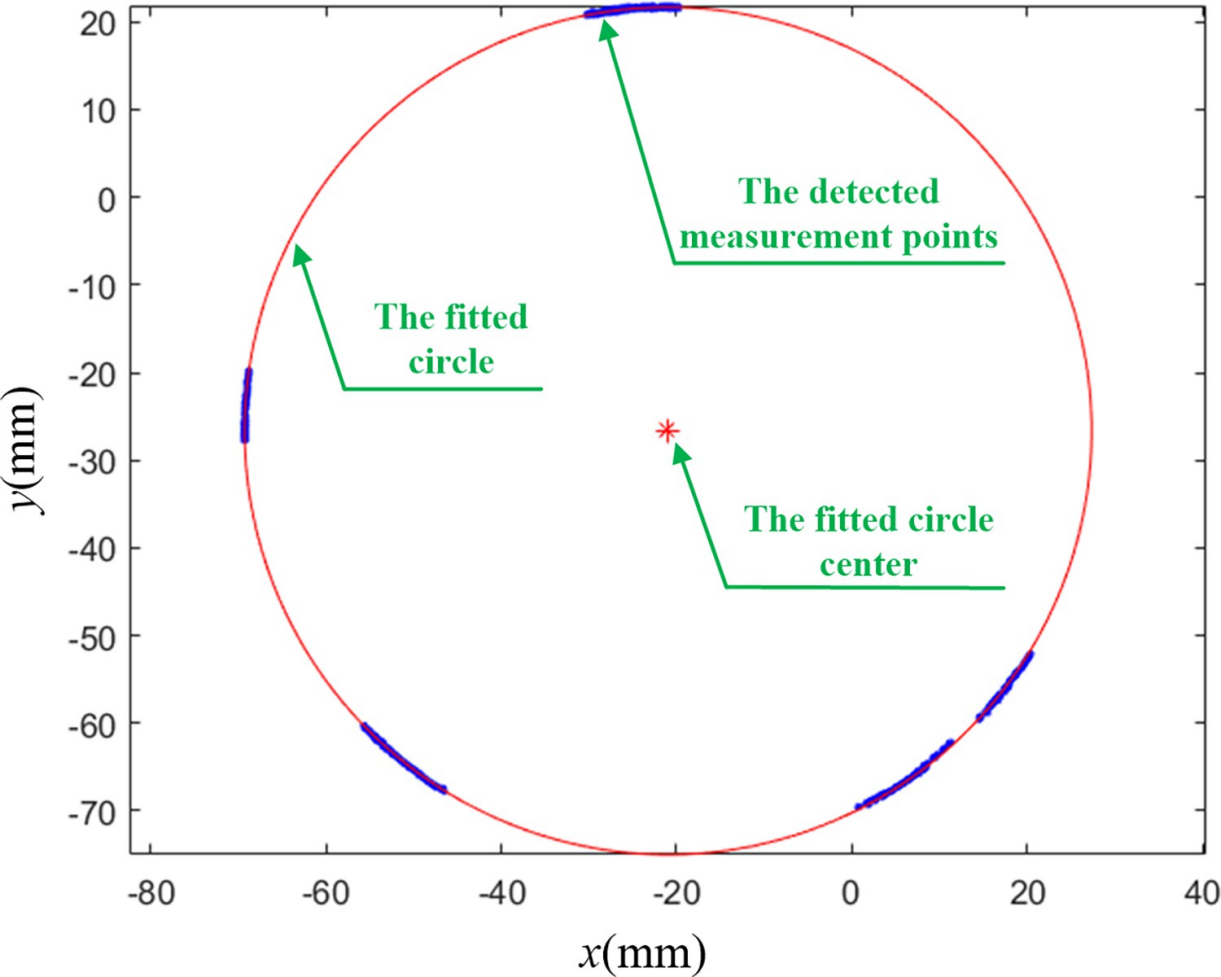
An example of circle geometric fitting.

According to the method in this article, the Matlab software is used to complete online measurement for dimensions of disk parts based on machine vision. In the world coordinate system, the inner and outer diameters of disk parts are measured by geometrically fitting the part edge circle. Center and radius of the circle can be calculated by algebraic fitting used Matlab firstly, and the results are used as the initial values for geometric fitting. Then, the Gaussian Newton algorithm [24] is used to iteratively calculate the center and radius according to Eqs (12) and (18) to complete the circle geometric fitting. The duration for executing the main experimental program is approximately 5 seconds. An example of the fitting results is shown in Fig 5.

The measurement results of inner, outer diameters and thickness of disk parts are shown in Table 3. In Table 3, "measurement values" are the average values of 10 times measurement results by the method in this work, "reference values" are the part dimensions in Table 1, "errors" are deviations between measurement values and reference values, "absolute errors" are absolute values of errors, "standard deviations" are the standard deviations of 10 times measurement results.

**Table 3:** Measurement results and comparison of disk parts dimensions(mm).

Disk Parts		Measurement values	Comparison values	Reference values	Comparison errors	Errors	Absolute errors	Standard deviations
**Pulley**	**Inner diameter (*d***_***n***_ **)**	13.004	13.026	13.016	0.010	-0.012	0.012	0.003
	**Outer diameter (*d***_***w***_ **)**	63.143	63.147	63.126	0.021	0.017	0.017	0.008
	**Thickness (*L***_***H***_ **)**	19.952	/	19.961	/	-0.009	0.009	0.004
**Flange**	**Inner diameter (*d***_***n***_ **)**	32.535	32.532	32.518	0.014	0.017	0.017	0.003
	**Outer diameter (*d***_***w***_ **)**	99.276	99.307	99.294	0.013	-0.018	0.018	0.006
	**Thickness (*L***_***H***_ **)**	13.536	/	13.525	/	0.011	0.011	0.004
**Gear**	**Inner diameter (*d***_***n***_ **)**	31.637	31.639	31.620	0.019	0.017	0.017	0.006
	**Outer diameter (*d***_***w***_ **)**	113.341	113.339	113.360	-0.021	-0.019	0.019	0.009
	**Thickness (*L***_***n***_ **)**	27.479	/	27.463	/	0.016	0.016	0.007

Commonly used visual measurement for dimensions of disk parts currently is realized by the measuring projector, with a telecentric lens, which has high accuracy but a small field of view and is difficult to measure the thickness of the disk part. The "comparison values" in Table 3 are the measurement results by measuring projector JVP400 (≤3+L/200(μm)), and "comparison errors" are deviations between comparison values and reference values.

Measurement values by the method have good agreement with the reference values and comparison values, as shown in Table 3. Compared to the measuring projector with telecentric lenses, standard industrial camera features low cost and good universality, and the thickness of disk parts is difficult to be measured by the measuring projector. From the table, measurement accuracy of the method can meet the requirements for level IT7 tolerance of hole and shaft dimensions in GB/T 1800.1–2020 (eqv ISO 286–1: 2010). The measurement range of the dimensions of disk parts completed by the method is as follows: the inner diameters are in 13mm-34mm, the outer diameters are in 62mm-114mm and the thicknesses are in 14mm-28mm. The influence of projection distortion and chamfer on the measurement is effectively reduced, concluding that the method is correct and accurate. Then the detected errors of pixel coordinates of the measurement points are critical factors affecting the accuracy. The errors are mainly caused by image quality and detection methods. Improving the detection accuracy of measurement points is the main research direction in the future.

## 5 Conclusion

This work proposes an online measurement method for dimensions of disk parts based on machine vision just by a single image. In the method, the technical way of reducing the influence of projection distortion and chamfer is effective. The method is suitable for multiple types of disk parts, meeting the requirements for inner, outer diameters and thicknesses of universal grade disk parts. The detected accuracy of measurement points on the image is the main factor affecting the measurement accuracy.

## Supporting information

S1 File The minimum data set used in the study. (DOC)

S1 Appendix (DOC)
