# Supplementary material for: Online measurement method for dimensions of disk parts based on machine vision
Source: PLoS One. 2024 Jul 25;19(7):e0307525. doi: 10.1371/journal.pone.0307525 (PMC11271902; doi:10.1371/journal.pone.0307525)
Supplement: S1 File — (DOC) [file pone.0307525.s001.doc]

The minimum data set used in the study.

1.The values behind the means, standard deviations.

| **No.** | **Pulley** | | | **Flange** | | | **Gear** | | |
| --- | --- | --- | --- | --- | --- | --- | --- | --- | --- |
|  | **Inner Diameters** | **Outer Diameters** | **Thicknesses** | **Inner Diameters** | **Outer Diameters** | **Thicknesses** | **Inner Diameters** | **Outer Diameters** | **Thicknesses** |
| **1** | 13.009 | 63.129 | 19.951 | 32.539 | 99.267 | 13.529 | 31.639 | 113.352 | 27.473 |
| **2** | 13.001 | 63.136 | 19.956 | 32.534 | 99.266 | 13.531 | 31.627 | 113.343 | 27.486 |
| **3** | 13.006 | 63.142 | 19.946 | 32.534 | 99.279 | 13.532 | 31.639 | 113.349 | 27.482 |
| **4** | 12.998 | 63.133 | 19.952 | 32.533 | 99.283 | 13.537 | 31.626 | 113.333 | 27.470 |
| **5** | 13.004 | 63.142 | 19.959 | 32.536 | 99.269 | 13.534 | 31.637 | 113.346 | 27.469 |
| **6** | 13.007 | 63.151 | 19.953 | 32.537 | 99.281 | 13.538 | 31.645 | 113.335 | 27.474 |
| **7** | 13.005 | 63.144 | 19.956 | 32.534 | 99.274 | 13.537 | 31.640 | 113.338 | 27.483 |
| **8** | 13.001 | 63.151 | 19.947 | 32.539 | 99.276 | 13.542 | 31.642 | 113.355 | 27.489 |
| **9** | 13.004 | 63.149 | 19.946 | 32.531 | 99.279 | 13.544 | 31.638 | 113.332 | 27.476 |
| **10** | 13.005 | 63.155 | 19.949 | 32.529 | 99.282 | 13.537 | 31.639 | 113.328 | 27.483 |
| **Means** | 13.004 | 63.143 | 19.952 | 32.535 | 99.276 | 13.536 | 31.637 | 113.341 | 27.479 |
| **Standard deviations** | 0.003 | 0.008 | 0.004 | 0.003 | 0.006 | 0.004 | 0.006 | 0.009 | 0.007 |

2.The points extracted from images for analysis and building the graph

**1)Pulley (pixel)**

*u* coordinates:

1286.03 1287.15 1289.01 1291.10 1293.08 1294.94 1294.98 1296.05 1297.12 1299.15 1299.97 1301.16 1302.02 1304.00 1305.07 1306.03 1307.05 1307.86 1309.06 1310.21 1311.10 1311.92 1312.88 1314.87 1316.11 1318.03 1320.96 1322.83 1339.07 1342.15 1345.00 1346.95 1348.03 1348.92 1350.01 1351.02 1352.27 1353.24 1354.03 1355.95 1357.05 1358.11 1358.88 1359.94 1360.90 1362.02 1363.10 1364.96 1366.07 1367.09 1367.84 1368.89 1370.05 1370.94 1372.03 1373.13 1374.27 1375.98 1377.86 1380.00 1382.07 1384.81 1387.08 1554.02 1556.01 1558.10 1560.04 1562.06 1564.01 1565.99 1568.04 1569.96 1572.00 1574.11 1574.98 1578.12 1580.05 1580.99 1583.03 1584.01 1585.99 1588.00 1590.98 1593.01 1595.08 1595.95 1598.03 1600.01 1602.12 1604.06 1605.04 1607.00 2055.01 2057.99 2060.00 2062.01 2064.99 2067.01 2069.05 2071.08 2073.06 2075.98 2079.00 2083.96 2087.80 2091.13 2094.04 2096.99 2098.98 2100.92 2102.00 2103.99 2106.93 2107.94 2419.08 2420.97 2422.97 2426.05 2427.07 2429.13 2431.11 2432.06 2433.03 2434.98 2435.83 2436.87 2438.13 2439.03 2440.94 2440.99 2441.95 2443.10 2444.13 2445.06 2446.02 2447.98 2448.95 2449.97 2450.11 2451.00 2452.66 2455.09 2456.80 2459.12 2461.93 2463.59 2464.94 2466.96 2509.05 2510.06 2511.93 2513.03 2514.07 2516.18 2517.93 2518.02 2520.12 2521.19 2521.78 2523.82 2524.08 2524.03 2525.04 2526.88 2527.90 2529.83 2530.09 2531.87 2532.77 2535.06 2535.91 2536.08 2536.86 2546.03 2545.99 2546.08 2546.02 2546.07 2546.05 2546.08 2546.04 2546.05 2546.99 2546.97 2557.99 2558.99 2559.03 2560.02 2559.91 2559.86 2559.97 2559.91 2559.82 2559.98 2560.90 2561.07 2487.13 2488.08 2491.03 2493.05 2495.01 2496.97 2498.04 2499.12 2501.15 2502.11 2502.06 2502.99 2503.95 2505.05 2506.01 2505.91 2507.78 2508.89 2511.91 2514.00 2516.03 2517.07 2517.98 2518.92 2520.96 2522.99 2178.02 2180.01 2182.04 2183.89 2185.85 2188.01 2190.03 2192.05 2193.97 2197.96 2198.86 2200.96 2202.73 2203.88 2205.93 2207.99 2209.03 2211.03 2213.05 2215.02 2216.85 2218.91 2222.01 2226.07 1516.13 1517.08 1519.06 1521.04 1522.06 1524.05 1525.00 1527.03 1529.10 1531.04 1532.95 1534.12 1536.07 1538.88 1539.93 1542.11 1543.97 1544.94 1547.00 1549.01 1550.07 1551.81 1552.99 1554.13 1556.02 1558.02 1560.00 1561.97 1563.06 1565.02 1565.93 1568.99

*v* coordinates:

1142.01 1142.07 1134.00 1130.05 1126.05 1119.98 1142.99 1141.02 1139.06 1114.10 1131.99 1130.08 1128.02 1105.00 1104.04 1102.02 1102.03 1115.95 1114.03 1097.10 1094.07 1108.94 1107.91 1103.95 1104.05 1099.02 1093.97 1088.91 1047.04 1045.12 1039.00 1035.97 1034.02 1030.95 1030.00 1028.01 1026.20 1026.19 1041.02 1022.96 1021.03 1020.07 1032.92 1031.96 1029.93 1029.02 1028.09 1009.97 1010.06 1009.06 1022.89 1021.94 1021.03 1017.97 1017.02 1016.07 1014.16 995.99 1007.89 1006.00 1004.05 997.87 997.06 855.03 853.01 841.17 840.07 839.12 837.02 835.98 835.07 833.94 833.00 833.15 844.96 830.19 829.10 841.98 827.08 840.02 838.98 837.01 822.96 822.02 821.16 834.91 820.06 819.02 818.20 817.13 830.09 828.99 780.94 770.05 771.01 782.87 772.03 772.96 784.90 784.83 785.78 777.05 786.91 787.40 788.32 789.86 791.83 780.03 792.09 782.27 793.88 794.14 784.15 794.14 1021.95 1026.02 1029.02 1031.97 1035.95 1038.89 1041.91 1043.95 1044.97 1025.02 1026.16 1029.08 1050.91 1052.98 1033.04 1058.01 1060.03 1060.93 1061.91 1062.96 1063.99 1044.02 1045.04 1046.02 1072.93 1075.00 1050.15 1053.93 1057.14 1060.90 1063.10 1066.15 1071.03 1074.02 1192.98 1196.98 1201.02 1205.99 1210.98 1215.95 1221.02 1226.00 1228.97 1189.93 1192.11 1193.08 1237.98 1242.99 1199.99 1203.05 1208.05 1213.04 1217.97 1221.06 1226.06 1229.98 1235.01 1239.99 1244.06 1396.00 1401.00 1406.00 1411.00 1418.00 1422.99 1432.00 1443.00 1448.00 1429.00 1435.00 1447.00 1397.00 1422.00 1403.00 1408.00 1413.00 1423.00 1428.00 1437.99 1443.00 1431.00 1436.01 1686.07 1681.04 1672.01 1670.02 1664.01 1656.99 1654.01 1653.04 1648.06 1683.04 1691.02 1686.99 1679.97 1640.02 1637.00 1682.96 1672.91 1672.96 1666.95 1664.00 1658.01 1657.02 1648.99 1643.96 1641.98 1637.99 1999.05 1999.02 1998.10 2015.70 2014.68 1995.02 1993.04 1992.08 2010.92 2005.83 2009.66 2005.88 2002.63 2002.85 2000.88 1983.99 2001.07 1982.06 1981.08 1980.03 1994.74 1993.83 1976.02 1975.11 1949.80 1966.83 1967.89 1952.95 1968.91 1969.93 1972.00 1957.95 1974.83 1974.93 1961.08 1977.82 1978.88 1964.20 1980.10 1980.86 1968.05 1984.13 1985.00 1970.99 1986.87 1974.26 1990.01 1991.84 1976.97 1976.95 1978.01 1978.08 1994.88 1979.97 1998.15 1983.01

**2)Flange (pixel)**

*u* coordinates:

2352.04 2372.95 2377.02 2379.87 2386.93 2391.98 2395.05 2397.99 2409.98 2412.85 2416.03 2417.96 2421.91 2426.99 2432.01 2437.05 2446.02 2451.00 2457.00 2462.05 2467.01 2477.03 2482.04 2490.09 2495.04 2499.98 2503.02 2507.03 2512.05 2514.97 2516.90 2520.12 2522.01 2539.04 2544.02 2552.95 2568.98 2581.97 2657.09 2658.98 2673.11 2681.97 2686.02 2692.01 2695.02 2697.96 2704.13 2705.02 2709.02 2711.08 2721.05 2724.09 2728.11 2733.06 2735.94 2745.05 2747.92 2750.03 2752.97 2762.99 2765.85 2770.02 2774.01 2776.87 2780.97 2783.88 2786.98 1669.98 1673.00 1679.00 1681.99 1684.99 1686.99 1690.00 1693.01 1694.98 1698.01 1703.00 1705.98 1709.99 1711.98 1714.99 1718.99 1721.99 1726.01 1726.99 1728.99 1732.00 1733.99 1738.00 1739.99 1743.00 1745.98 1749.99 1754.00 1757.99 1759.98 1761.99 1763.98 1767.99 1770.01 1773.00 1774.99 1777.99 1781.01 1783.99 1785.99 1789.01 1793.00 1794.99 1798.00 1801.00 1804.00 1808.00 1810.00 1812.00 1815.01 1817.00 1820.00 1824.00 1828.00 1831.01 1834.00 1836.00 1838.00 1842.00 1845.00 1849.99 1854.99 1856.99 1861.00 1865.99 1868.99 1871.99 1875.00 1877.00 1879.00 1881.00 1883.00 1886.00 1891.00 1894.00 1897.00 1899.00 806.75 806.85 806.72 807.96 807.94 808.00 807.81 808.99 808.91 808.86 808.82 809.91 809.97 810.03 809.97 810.17 810.13 809.80 811.09 811.09 811.05 811.14 811.01 811.12 811.04 810.98 810.90 812.02 812.13 813.19 814.10 814.24 814.18 813.90 815.09 814.97 815.11 816.07 816.03 817.14 818.23 817.99 819.04 1111.21 1113.99 1120.09 1124.15 1129.07 1134.05 1137.87 1139.95 1141.95 1143.81 1145.91 1148.09 1153.01 1156.15 1158.24 1159.97 1164.03 1166.96 1169.03 1172.02 1177.00 1180.04 1184.08 1189.05 1194.04 1199.09 1204.01 1208.99 1213.93 1219.05 1224.02 1228.03 1233.06 1238.01 1243.09 1250.10 1252.92 1258.02 1262.99 1268.05 1273.03 1278.00 1283.10 1287.99 1292.98 1297.98 1303.00 1307.99 1311.09

2355.99 2361.89 2375.09 2380.08 2385.90 2392.09 2409.14 2413.87 2418.87 2426.05 2431.14 2436.09 2440.90 2450.99 2455.95 2460.94 2466.11 2470.98 2476.03 2481.88 2486.99 2491.88 2497.05 2502.13 2516.92 2526.94 2532.00 2537.03 2541.97 2626.21 2631.95 2635.93 2641.03 2643.87 2648.98 2650.97 2653.94 2655.04 2657.94 2660.03 2662.97 2665.96 2667.91 2670.04 2673.11 2674.85 2675.97 2679.12 2683.05 2685.95 2689.12 2692.02 2706.98 2711.02 2712.92 2715.90 2719.84 2721.96 2724.91 2730.03 2730.93 2733.93 2737.02 2740.04 2742.13 2744.01 2747.08 2749.99 2754.16 2757.09 2760.10 1668.00 1673.01 1679.02 1683.01 1687.03 1690.02 1695.03 1699.01 1703.02 1713.01 1719.00 1753.02 1758.01 1763.01 1768.01 1773.01 1781.02 1798.01 1801.00 1805.01 1809.01 1812.02 1815.00 1818.01 1821.01 1824.02 1826.02 1830.01 1896.00 1900.00 822.84 822.97 823.10 822.86 822.94 822.83 824.07 824.02 823.80 824.89 827.04 827.08 826.78 826.91 826.79 827.97 827.92 829.09 829.02 828.78 830.15 829.72 831.31 831.06 830.91 832.06 831.86 832.71 834.30 833.97 834.88 836.41 835.93 835.88 836.99 1133.04 1137.99 1143.05 1148.99 1154.00 1158.94 1164.02 1168.85 1172.87 1177.90 1183.02 1188.03 1193.15 1197.84 1203.01 1207.99 1212.95 1218.03 1223.01 1228.09 1232.95 1238.00 1242.94 1247.94 1257.93 1263.02 1267.95 1272.89 1278.10 1282.94 1288.08 1292.93 1297.88 1302.95 1308.02 1312.99 1318.90 1323.97

*v* coordinates:

525.92 536.10 537.95 540.26 544.12 546.03 542.89 549.02 556.05 558.31 554.94 560.07 562.18 563.02 564.99 567.93 573.96 578.00 581.00 584.91 587.99 593.96 597.94 603.88 607.95 612.03 615.98 615.95 617.94 626.03 628.11 624.86 631.99 645.94 649.98 658.05 672.02 686.04 744.90 751.02 763.92 775.02 779.98 785.99 789.98 795.03 792.89 801.98 805.98 808.94 819.96 822.93 822.92 836.94 842.04 850.96 858.09 857.98 863.03 872.01 877.11 881.99 888.00 894.09 899.02 904.07 909.01 2518.10 2514.01 2515.02 2523.20 2519.04 2519.04 2515.97 2515.96 2520.11 2517.91 2518.00 2521.08 2520.07 2525.08 2526.08 2529.06 2523.08 2520.94 2528.10 2528.13 2524.03 2529.06 2523.05 2531.14 2526.00 2531.16 2526.08 2526.02 2527.07 2529.14 2527.03 2532.13 2527.04 2526.95 2527.98 2533.10 2529.03 2529.92 2535.06 2535.09 2529.90 2532.05 2535.13 2531.05 2534.03 2533.13 2531.95 2536.05 2537.09 2532.05 2536.05 2532.08 2532.96 2534.01 2533.08 2535.13 2535.09 2534.14 2530.92 2533.10 2533.10 2533.10 2536.10 2535.05 2533.05 2534.05 2537.09 2533.01 2535.05 2536.06 2535.05 2532.98 2534.00 2535.08 2531.95 2534.04 2537.07 1443.97 1462.00 1487.01 1451.00 1463.00 1484.00 1511.01 1509.00 1516.01 1521.02 1536.01 1446.99 1456.00 1466.00 1477.00 1497.01 1502.01 1540.01 1451.01 1468.00 1474.00 1482.00 1490.00 1524.99 1530.00 1545.00 1555.01 1540.00 1548.99 1538.00 1555.99 1561.00 1565.98 1588.01 1570.99 1579.00 1583.99 1590.99 1596.00 1600.98 1605.97 1611.00 1614.99 730.20 725.99 721.10 717.15 712.08 707.07 699.86 704.94 700.96 692.81 692.91 696.09 690.01 692.16 689.21 683.97 680.03 674.95 675.03 673.02 668.00 665.05 662.09 657.07 653.04 649.12 645.01 639.99 635.91 633.07 629.03 626.04 623.09 619.01 616.12 611.15 605.90 603.02 598.99 596.07 592.04 587.99 586.17 581.98 577.97 576.94 574.01 570.98 574.18

539.02 543.17 549.84 552.85 557.17 559.84 569.78 574.21 577.21 580.93 583.79 586.86 591.15 597.02 601.07 604.08 606.84 611.03 613.95 619.17 622.02 626.17 628.92 631.84 644.11 652.09 656.00 658.96 664.03 735.83 743.05 746.05 749.98 754.11 758.02 759.03 761.07 760.96 764.05 768.98 773.03 776.04 779.07 783.97 779.87 789.12 793.03 789.89 795.94 802.05 801.87 808.98 827.02 830.98 836.07 838.07 844.11 849.03 854.06 855.98 861.04 866.04 865.99 870.98 875.94 881.00 885.95 891.01 895.89 900.94 905.94 2494.00 2494.93 2497.89 2494.89 2494.86 2498.88 2497.82 2499.92 2498.88 2499.96 2507.96 2504.77 2506.89 2506.87 2508.86 2508.87 2506.76 2509.92 2512.88 2510.81 2511.92 2513.85 2513.97 2511.93 2514.94 2511.88 2515.81 2513.84 2512.91 2511.83 1453.00 1458.00 1462.99 1468.02 1473.00 1480.01 1445.99 1479.00 1487.03 1492.03 1496.99 1502.00 1507.01 1512.00 1517.03 1522.00 1527.01 1531.98 1537.00 1542.02 1546.98 1552.03 1557.01 1561.99 1567.01 1572.00 1577.02 1582.02 1586.99 1592.01 1597.02 1601.96 1607.01 1612.01 1617.00 729.04 723.99 720.05 713.98 710.00 704.93 701.02 695.83 691.87 687.89 684.02 680.04 676.17 670.81 667.01 662.99 658.94 655.03 651.01 647.11 642.93 639.00 634.92 630.92 623.90 621.03 616.93 612.86 610.13 605.92 603.13 598.91 594.82 591.93 589.02 584.99 579.88 576.95

**3)Gear (pixel)**

*u* coordinates:

921.57 922.28 921.79 923.15 923.10 923.16 923.05 923.95 933.77 934.36 934.88 934.95 961.11 961.02 961.84 964.02 966.16 967.97 1002.22 1004.80 1007.05 1007.77 1008.83 1010.07 1010.98 1012.95 1014.00 1065.00 1065.09 1065.94 1067.17 1067.89 1069.80 1086.04 1032.20 1033.42 1033.02 1034.99 1036.88 1038.06 1039.95 1041.06 1042.88 1043.88 927.02 927.12 927.15 926.69 927.87 933.16 945.58 946.93 948.08 948.72 949.86 952.23 953.97 956.88 961.02 980.91 983.12 985.90 988.16 988.89 991.10 993.07 996.97 3207.04 3213.15 3216.99 3218.97 3223.06 3225.09 3227.04 3229.05 3280.06 3281.17 3284.05 3286.03 3289.04 3290.06 3386.17 3387.08 3387.16 3386.97 3387.08 3388.11 3388.11 3034.99 3038.06 3040.99 3043.03 3045.06 3048.03 3051.03 3053.04 3056.02 3058.12 3060.97 3063.10 2824.01 2834.04 2838.03 2841.01 2843.14 2848.09 1032.98 1034.93 1041.04 1044.16 1047.98 1055.00 1102.14 1104.05 1105.96 1107.88 1115.80 1116.61 1119.19 1121.92 1178.17 1179.34 1181.74 1185.02 1185.93 1187.82 1189.03 1190.86 1191.88 1192.99 1193.81 1194.72 1197.04 1199.01 1202.97 1205.09 1208.07 3321.99 3325.94 3328.00 3330.02 3331.05 3332.99 3334.11 3336.01 3337.02 3339.01 3361.14 3366.19 3365.98 3367.18 3367.05 3368.06 3369.15 3370.97 3372.00 3239.16 3242.91 3244.17 3248.02 3249.91

*v* coordinates:

1626.92 1632.01 1637.03 1622.01 1627.02 1642.99 1647.99 1651.03 1497.94 1502.94 1493.97 1508.01 1362.04 1367.00 1358.93 1354.00 1346.02 1337.99 1231.20 1226.90 1218.03 1225.93 1225.95 1224.02 1221.99 1207.99 1208.00 1099.00 1104.02 1089.98 1086.07 1083.92 1080.88 1114.02 1238.07 1237.21 1252.00 1244.00 1238.94 1238.03 1229.98 1229.02 1221.95 1219.96 1765.00 1770.00 1774.99 1780.05 1784.03 1792.75 1908.12 1912.00 1916.98 1922.05 1927.04 1930.90 1934.04 1936.21 1936.95 2039.02 2042.96 2055.04 2057.94 2062.05 2065.95 2068.91 2071.13 2299.03 2293.11 2287.99 2283.98 2278.03 2274.04 2271.02 2267.04 2166.03 2164.10 2161.03 2157.01 2148.02 2147.02 1597.99 1602.99 1608.00 1613.00 1624.99 1618.00 1627.98 2521.96 2519.08 2514.98 2513.03 2511.07 2509.03 2505.03 2504.04 2500.01 2499.12 2494.97 2494.09 2691.01 2684.07 2682.05 2679.01 2678.18 2675.12 2172.01 2177.03 2189.99 2194.82 2197.09 2199.00 2304.92 2308.96 2312.04 2315.08 2324.33 2330.09 2324.56 2335.47 2420.00 2423.73 2426.30 2427.99 2430.07 2420.11 2432.96 2424.08 2426.11 2427.01 2429.19 2430.24 2440.88 2440.87 2435.91 2436.19 2435.24 2045.99 2047.95 2045.00 2037.01 2037.02 2033.00 2028.04 2023.00 2021.00 2016.00 1902.00 1894.02 1907.99 1896.00 1901.01 1887.03 1886.06 1882.99 1880.00 1081.88 1084.04 1088.96 1095.99 1101.02
