# Supplementary material for: Online measurement method for dimensions of disk parts based on machine vision
Source: PLoS One. 2024 Jul 25;19(7):e0307525. doi: 10.1371/journal.pone.0307525 (PMC11271902; doi:10.1371/journal.pone.0307525)
Supplement: S1 Appendix — (DOC) [file pone.0307525.s002.doc]

**Appendix A**

The calibration results of camera intrinsic parameters are shown in Table A1.

**Table A1. The calibration results of camera intrinsic parameters.**

| $\alpha$ | $\beta$ | $\gamma$ | $u_{0}$ | $v_{0}$ | $k_{1}$ | $k_{2}$ | $p_{1}$ | $p_{2}$ |
| --- | --- | --- | --- | --- | --- | --- | --- | --- |
| 14015.71 | 14009.61 | 7.08 | 2096.75 | 1435.22 | -0.1690 | 11.9893 | 0.0003 | -0.0024 |

In camera coordinate system, the calibration result of calibration board plane equation is

$-0.0179x-0.0968y-1.5192z+1000=0$ (A1)

In the camera coordinate system, the thickness of checkerboard calibration board used in the experiment is 5.44mm, and then calculation result of the equation for workbench plane (that is lower end face of the part) is

$-0.0179x-0.0968y-1.5192z+1008.2820=0$ (A2)

**Appendix B**

The transformation matrix $\mathbf{I}$ between camera coordinate system and world coordinate system is

$\mathbf{I}=\left[\begin{matrix} 0.9999 & \text{-0.0004} & 0.0117 \\ \text{-0.0004} & 0.9980 & 0.0636 \\ \text{-0.0117} & \text{-0.0636} & 0.9979 \end{matrix}\right]$ (B1)

Camera coordinates of the intersection $P_{0}$ between $z$-axis of camera coordinate system and the part lower end face are calculated by equation (A2), and then the $Z$ world coordinate $Z_{0}$ of the point on lower end face are calculated, as shown in Table B1.

**Table B1. The $\mathbf{Z}$ world coordinate $\mathbf{Z}_{\mathbf{0}}$ of the point on lower end face.**

| **Camera coordinates of** $\mathbf{P}_{\mathbf{0}}$ | $\mathbf{Z}_{\mathbf{0}}$ |
| --- | --- |
| (0,0, 663.6766) | 662.2872 |
